# A time function-based prediction model of mining subsidence: application to the Barapukuria coal mine, Bangla

**DOI:** 10.1038/s41598-022-23303-9

**Published:** 2022-11-01

**Authors:** Xingsheng Zhang, Shaobin Yan, Haicheng Tan, Jinyu Dong

**Affiliations:** School of Earth Sciences and Engineering, North China University of Water Resources and Electric Power, Zhengzhou, 450046 China

**Keywords:** Natural hazards, Solid Earth sciences

## Abstract

Coal mining may lead to ground subsidence in a long term and is widely distributed, which can cause environmental damage and other disasters. Predicting the dynamic process of ground subsidence in real time is very important for offering theoretical or technical guidance to deal with the consequences of mining. In this study, we developed a prediction method for dynamic ground subsidence using a time function model that considers two stages of surface subsidence and reflects the law of surface subsidence in goaf. We applied the model to the Barapukuria mine, and our simulation shows that the prediction results are in good agreement with the monitoring data. Our results suggest that the dynamic development of the ground subsidence basin may be an effective measure to assess the loss of ground and provide early warning of oncoming hazards.

## Introduction

Coal mining often leads to ground subsidence around mining areas, which can cause problems, such as water accumulation, agricultural reduction, road collapse, and deformation or damage to buildings^1,2^. Underground mining causes the ground surface to move downward relative to the initial mining position, which promotes subsidence basin^3–6^. Surface subsidence is a dynamic process that gradually develops with the advancement of the mining working face^7–9^. An arbitrary point on the ground surface continues to subside as extraction is performed within a critical area below that point^10^. It is important to predict the dynamic development of a surface over time.

The prediction of mining subsidence is an important issue for the mining industry, government, and the affected people. This is particularly important for towns and engineering projects where mining occurs^11^. Based on the analysis of subsidence monitoring data, influential functions can be used to describe the influence of the excavation elementary part of the formation subsidence^12–14^. The dynamic prediction of mining subsidence can be obtained by multiplying the static prediction results with the corresponding time coefficient^15,16^. Double-parameter time functions and prediction parameters have been proposed to improve simulation performance^17–20^. Although the Knothe time function can be used to predict the dynamic movement and deformation of the ground surface, it is still difficult to determine the coefficient of the influence function because land subsidence is affected by geological parameters and geomechanical mechanisms.

In this paper, we first present the major contribution of this study—a prediction model developed with the parameters of ground subsidence. Then, with the help of MATLAB programming platform, we simulated the surface subsidence process over time in the Barapukuria mining area in Bangladesh. The results show that the numerical simulation matches the monitoring data well. We hope that our study offers a dynamic prediction of ground subsidence and provides a basis for the early warning of forthcoming hazards.

## Subsidence mechanism of coal seam mining

**Figure 1:**
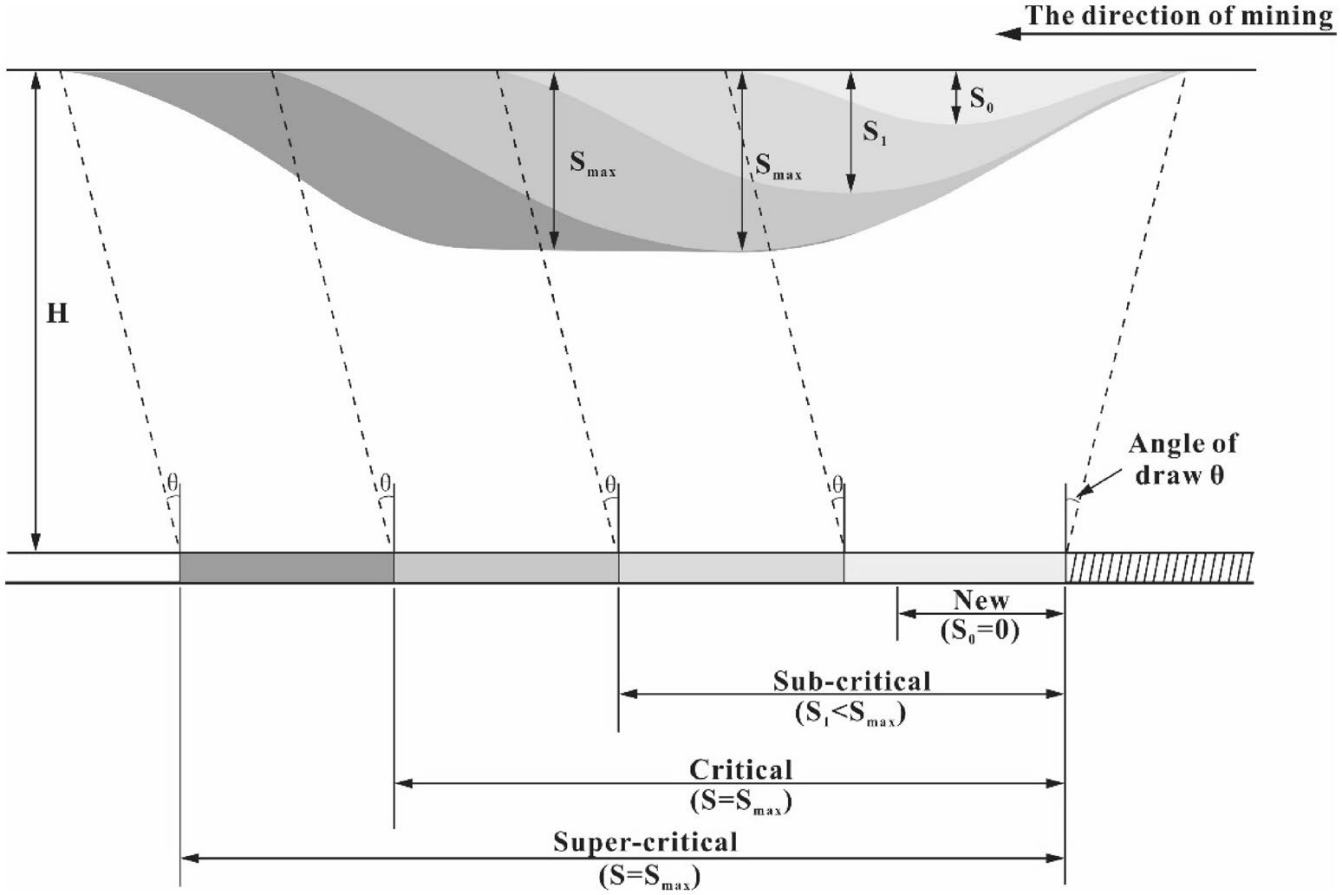
The vertical section sketch of coal seam mining subsidence.

The mechanism of ground movement caused by mining is complex and influenced by the geological environment of the mine and overlying strata. Although an accurate model is difficult to find, an approximate prediction model can also be used to forecast subsidence caused by mining by modifying certain influence parameters. In the process of mining, ground subsidence continues to increase and forms a subsidence basin, as shown in Fig. 1 (vertical section), which indicates that the value of the subsidence and the influence range continue to expand from right to left (along the direction of mining). The subsidence will reach a stable maximum value when the coal seam reaches full extraction.

### Spatial influence function

The spatial influence function can predict ground subsidence, which should not only take into account the main characteristics such as the depth of the coal seam, maximum possible subsidence, and time factor, but also involve the evolution of subsidence in space. Here, we introduce a spatial influence function^21^, the formula is expressed as follows:

1 $$w\left( x \right) = \frac{{S_{\max } }}{{\left( {1 + 4n} \right) \cdot \left( {kH} \right)^{2} }} \cdot \left[ {e^{{ - \pi \left( \frac{x}{kH} \right)^{2} }} + n \cdot e^{{ - \pi \left( \frac{x}{2kH} \right)^{2} }} } \right]$$

where $w\left( x \right)$ is the influence function that extends to all mining units of the coal seams so that the subsidence at point $x$ on the surface can be computed. $S_{\max }$ is the maximum possible subsidence. $H$ represents the depth of mining elements. $n$ and $k$ represent the shape and magnitude of the ground subsidence trough, respectively.

### Time influence function

The subsidence of the surface cannot be completed instantaneously after mining, and always develops over a period of time and even several years to reach a relatively stable state. One of the most widely used time functions proposed by Knothe^12^, assumes that the surface subsidence velocity $\partial W\left( t \right)/\partial t$ is proportional to the difference between the maximum surface subsidence $W_{0}$ and dynamic subsidence $W\left( t \right)$ at a given time $t$.

2 $$\frac{\partial W\left( t \right)}{{\partial t}} = c\left( {W_{0} - W\left( t \right)} \right)$$

where $c$ denotes the influence coefficient of the time factor related to the mechanical properties of the overlying strata. Combined with the initial time boundary conditions: $t = 0$ and $W\left( t \right) = 0$, by solving the differential equation, the relationship between surface subsidence and time can be obtained:

3 $$W\left( t \right) = W_{0} \left( {1 - e^{ - ct} } \right)$$

The above relationship represents the subsidence expression of the dynamic prediction of surface movement based on the Knothe time function. The accuracy of the Knothe time function depends mainly on parameter $c$. The smaller the $c$ value, the smaller the Knothe time function value. In contrast, the value of the Knothe time function increases as $c$ increases. In other words, it takes longer for surface subsidence to stabilize as the value of $c$ increases.

### Piecewise Knothe time function

**Figure 2:**
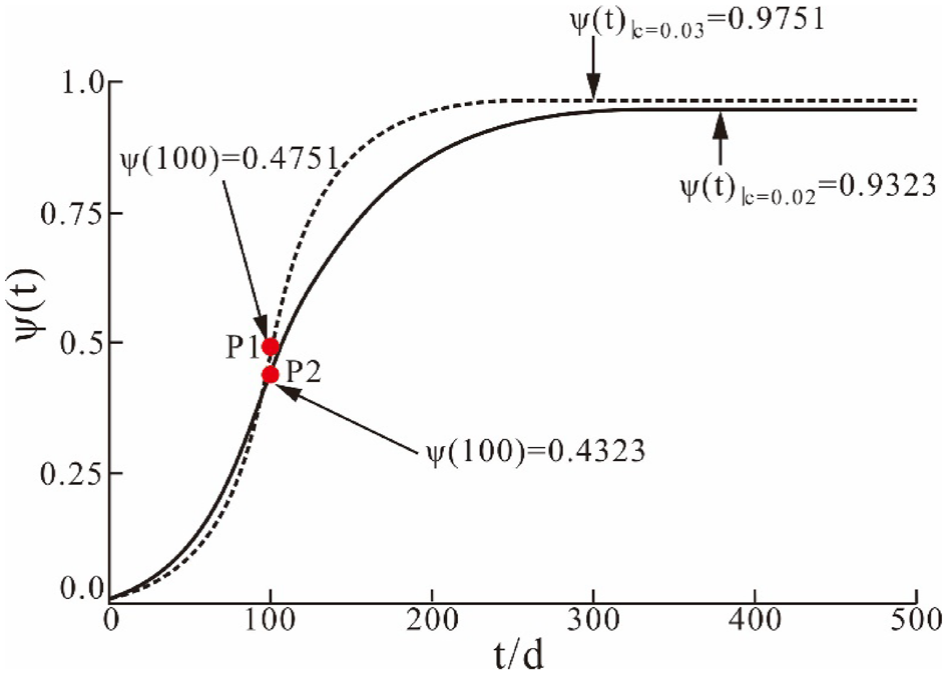
Piecewise Knothe time function graphic with $c$ at different values (P1, P2 are inflection points of the two curves).

To reflect the dynamic change law of surface subsidence, the Knothe time function is improved to meet the need for dynamic prediction of practical projects^22^. The improved function includes two functional expressions. First, the surface subsidence velocity gradually increases from zero to the maximum value, and the surface points gradually bend owing to mining. The second shows that the surface subsidence velocity decreases from the maximum to zero. The functional relationship of the subsidence velocity with time is as follows:

4 $$W(t) = \left\{ {\begin{array}{*{20}c} {0.5W_{k} \left[ {e^{ - c(\tau - t)} - e^{ - c\tau } } \right],} & {0 < t \le \tau } \\ {0.5W_{k} \left[ {2 - e^{ - c(t - \tau )} - e^{ - c\tau } } \right],} & {\tau < t \le T} \\ \end{array} } \right.$$

5 $$V_{t} = \left\{ {\begin{array}{*{20}c} {0.5cW_{k} e^{{ - c\left( {\tau - t} \right)}} ,} & {0 < t \le \tau } \\ {0.5cW_{k} e^{{ - c\left( {t - \tau } \right)}} ,} & {\tau < t \le T} \\ \end{array} } \right.$$

where $t$ is the moment of prediction, $W(t)$ is the subsidence value of the surface, $W_{1} \left( t \right)$ and $W_{2} \left( t \right)$ are the surface subsidence values of the first and the second stages, respectively, at moment $t$. $V_{t}$ is the subsidence velocity of the surface at moment $t$, $V_{1} \left( t \right)$ and $V_{2} \left( t \right)$ are the surface subsidence velocities of the first stage and second stage at the moment $t$, respectively. $W_{k}$ represents the final subsidence value of the surface, and $\tau$ is the moment when the maximum subsidence velocity of the surface subsidence occurs, which can also be regarded as the moment when the inflection point of the subsidence curve appears, where $T$ is the total subsidence time for the two stages (shown in Fig. 2).

For a near-horizontal coal seam, the surface subsidence reaches the maximum subsidence velocity $V_{\max }$ at time $\tau$. An inflection point exists in the surface subsidence curve, and the surface subsidence value $W_{\tau }$ is approximately equal to half of the maximum surface subsidence value $W_{\max }$. Thus, the theoretical value of the function is 0.5. As shown in Fig. 2, both final function values of $\psi (t)$ are close to, but cannot reach the value of 1, and the final function value decreases with a decrease in the $c$ value. Therefore, the piecewise time function for dynamic prediction does not match the final ground subsidence curve.

According to the above analysis, the theoretical subsidence value $W_{\tau }$ is not equal to half of the maximum surface subsidence value $W_{\max }$, and the final function value of $\psi (t)$ is close to 1, but cannot reach 1.

Here, an optimized piecewise Knothe time function^23^ is given as:

6 $$\varphi_{t} = \left\{ {\begin{array}{*{20}c} {\varphi_{1} \left( t \right) = \frac{1}{2}\left[ {\frac{{t - \tau \left( {1 - e^{ct} } \right)}}{{\tau e^{ct} }}} \right],} & {0 < t \le \tau } \\ {\varphi_{2} \left( t \right) = 1 - \frac{1}{2}e^{{c\left( {\tau - t} \right)}} ,} & {\tau < t \le T} \\ \end{array} } \right.$$

With the optimization of the time function, regardless of parameters $c$ and $\tau$, the function value is equal to the theoretical value at moment $\tau$, and the final time function value converges to 1, which can solve some theoretical problems of the original piecewise time function.

## The algorithm of surface subsidence prediction model

### Determination of prediction parameters

The prediction parameters for mining subsidence in the probability integral method have been calculated^24,25^. The coordinates of the monitoring points in the coordinate system of the working face are $\left( {x,y} \right)$, and the actual subsidence value is $z$. Using the least-squares method to fit the surface, after multiple iterations, a group of parameters can be obtained to minimize the square sum of the deviations between the fitted surface and the measured data. The mathematical model of the least-squares method is:

7 $$Q_{1} Q_{1}^{T} = \sum\limits_{k = 1}^{n} {\left[ {W_{zk} - W\left( {x,y} \right)} \right]^{2} } = \min$$

where $Q_{1}$ is the deviation between the measured subsidence at each monitoring point and the least-squares fitting value. $Q_{1}^{T}$ is the transpose of matrix $Q_{1}$. $k$ is the number of monitoring points, $W_{zk}$ is the measured subsidence deformation at each monitoring point, and $W\left( {x,y} \right)$ is the least-squares fitting value of the measured subsidence deformation at each monitoring point. According to the empirical value of the prediction parameters of the mining area, the initial value of the iteration is used for least-squares fitting to obtain the prediction parameters.

### Subsidence prediction method for the strike principal section

First, the mining area should be divided into *n*-equal units along the working face before the dynamic prediction of ground subsidence. The mining moment at the open-off cut and speed of the working face of the first mining unit are defined as the initial moments $t_{0}$ and $v_{1}$, respectively. The point of the open-off cut is considered as the coordinate origin, and the length of the first mining unit is $v_{1} t_{1}$. The excavation speed of the second unit is $v_{2}$, the length of which is $v_{2} t_{2}$, and the coordinate value of the terminal point of the second unit is $v_{1} t_{1} { + }v_{2} t_{2}$. The next mining units below can be obtained in this manner. The excavation speed of the *n*-*th* unit is $v_{n}$, the length of which is $v_{n} t_{n}$, and the coordinate value of the terminal point of the second unit is $v_{1} t_{1} { + }v_{2} t_{2} + \cdots v_{n} t_{n}$. The influence of each unit on the surface static subsidence when the inflection point displacement is considered in the next section.

The ground static subsidence of the first, second and *n*-*th* units induced after mining can be expressed as follows:

8 $$\left\{ \begin{aligned} W_{1} (x) = & W(x - s_{3} ) - W[(x - s_{3} - (v_{1} *t_{1} - s_{3} )] \, \\ \, = & W(x - s_{3} ) - W(x - v_{1} *t_{1} ) \\ W_{2} (x) = & W(x - v_{1} *t_{1} ) - W(x - v_{1} *t_{1} - v_{2} *t_{2} ) \\ W_{n} (x) = & W(x - v_{1} *t_{1} - \cdots - v_{n - 1} *t_{n - 1} ) - \\ \, & W[x - (v_{1} *t_{1} + v_{2} *t_{2} + \cdots + v_{n - 1} *t_{n - 1} + v_{n} *t_{n} + s_{4} )] \\ \end{aligned} \right.$$

Assuming that the predicted time of surface subsidence is $t$, the time function of surface subsidence induced by the first mining unit is $k_{1} (t)$, the time function of surface subsidence induced by the second mining unit is $k_{2} (t - t_{1} )$, and the time function of surface subsidence induced by the *n-th* mining unit is $k_{n} (t - t_{1} - \cdots t_{2} - \cdots t_{n - 1} )$. According to the superposition principle, it is necessary to multiply the time function corresponding to each mining unit by the corresponding static subsidence and then sum it to obtain the surface dynamic subsidence prediction formula, as follows:

9 $$\begin{gathered} W(x,t) = k(t)[W(x - s_{3} ) - W(x - v_{1} *t_{1} )] \hfill \\ + k(t - t_{1} )[W(x - v_{1} *t_{1} ) - W(x - v_{1} *t_{1} - v_{2} *t_{2} )] \hfill \\ + k(t - t_{1} - t_{2} )[W(x - v_{1} *t_{1} - v_{2} *t_{2} ) - W(x - v_{1} *t_{1} - v_{2} *t_{2} - v_{3} *t_{3} )] \hfill \\ + \cdots + k(t - t_{1} - t_{2} - \cdots t_{n} )[W(x - v_{1} *t_{1} - v_{2} *t_{2} - \cdots - v_{n - 1} *t_{n - 1} ) \hfill \\ - W(x - v_{1} *t_{1} - \cdots - v_{n - 1} *t_{n - 1} - v_{n} *t_{n} + s_{4} )] \hfill \\ \end{gathered}$$

In the actual mining process, the daily excavation speed and mining time remain constant: $v_{1} = v_{2} { = } \cdots { = }v_{n} ,{\kern 1pt} {\kern 1pt} {\kern 1pt} {\kern 1pt} {\kern 1pt} {\kern 1pt} t_{1} = t_{2} { = } \cdots { = }t_{n}$, and the dynamic subsidence prediction formula can be simplified as follows.

10 $$\begin{gathered} W(x,t) = k(t)[W(x - s_{3} ) - W(x - v_{d} *t_{d} )] \hfill \\ + k(t - t_{d} )[W(x - v_{d} *t_{d} ) - W(x - 2*v_{d} *t_{d} )] \hfill \\ + k(t - 2*t_{d} )[W(x - 2*v_{d} *t_{d} ) - W(x - 3*v_{d} *t_{d1} )] \hfill \\ + \cdots + k(t - (n - 1)t_{d} )[W(x - (n - 1)v_{d} *t_{d} ) - W(x - n*v_{d} *t_{d} + s_{4} )] \hfill \\ \end{gathered}$$

According to the above analysis, the formula for the probabilistic integral method is given.

11 $$W(x) = \frac{{W_{0} }}{2}\left[ {erf\left( {\frac{\sqrt \pi }{r}x} \right) + 1} \right]$$

where *r* is the major influence radius. When the predicted moment is greater than the total mining time, the mine was fully mined. We set $s_{3}$ and $s_{4}$ as zero if the displacement of the inflection point is ignored.

### Subsidence prediction method for the surface of mining area

**Figure 3:**
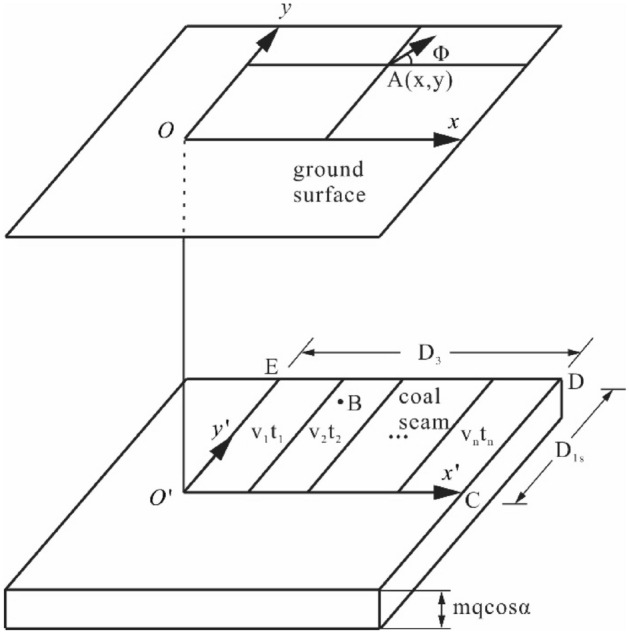
Influence of unit mining on arbitrary points on the surface.

During subsidence prediction process of the strike principal section, the subsidence of all the affected surface points can be predicted using the three-dimensional coordinate system, as shown in Fig. 3. The abscissa of the micro unit and an arbitrary point A on the surface are $x^{\prime}$ and $x$, respectively. Thus, the subsidence of point $A$ on the surface caused by this micro unit can be determined.

12 $$W_{A} \left( x \right) = \frac{1}{r}e^{{\frac{{ - \pi \left( {x - x^{\prime}} \right)^{2} }}{{r^{2} }}}}$$

To predict the subsidence of an arbitrary point on the surface of the mining area, a coal seam coordinate system is set with the origin and the $x^{\prime}$-axis parallel to the coal seam trend and the $y^{\prime}$-axis parallel to the coal seam inclination. The corresponding origin point of the surface coordinate system is set as $O$, and the x- and y-axes are parallel to the $x^{\prime}$- and $y^{\prime}$-axes, respectively, as shown in Fig. 3. If a micro unit $B$ with coordinates $\left( {x^{\prime},y^{\prime}} \right)$ is mined, its subsidence effect on point $A$ with surface coordinates $\left( {x,y} \right)$ can be expressed by the following equation:

13 $$W_{A} \left( {x,y} \right) = \frac{1}{r}e^{{ - \pi \frac{{\left( {x - x^{\prime}} \right)^{2} + \left( {y - y^{\prime}} \right)^{2} }}{{r^{2} }}}}$$

Figure 3 shows that the mining area is $EO_{1} CD$, the length of $ED$ is $D_{3}$, and the length of $CD$ is $D_{1s}$. Using Eq. (13), the surface subsidence caused by the mining area can be expressed as:

14 $$\begin{aligned} W\left( {x,y} \right) & = W_{0} \int_{0}^{{D_{3} }} {\int_{0}^{{D_{1s} }} {\frac{1}{{r^{2} }}} } e^{{ - \pi \frac{{\left( {x - x^{\prime}} \right)^{2} + \left( {y - y^{\prime}} \right)^{2} }}{{r^{2} }}}} dy^{\prime}dx^{\prime} \\ & = \frac{1}{{W_{0} }}\left[ {W\left( x \right) - W\left( {x - D_{3} } \right)} \right]\left[ {W\left( y \right) - W\left( {y - D_{1s} } \right)} \right] \\ & = \frac{1}{{W_{0} }}W_{0} \left( x \right)W_{0} \left( y \right) \\ \end{aligned}$$

where, $W_{0}$ represents the maximum value of the surface subsidence, which can be expressed by the empirical formula $W_{0}\,=\,mq\cos \alpha$, and $\alpha$ is the dip angle of the coal seam.

## Numerical Prediction

### Mining condition of Mumba coal mine

The mine is located in the northwest of Dhaka^26^, the capital of Bangladesh, with a geographical location of 25°31′–25°34′ N and 88°57′–88°59′ E. The spans of north–south and east–west are respectively about 4.9 km and 0.3 ~ 1.9 km about covering an area of 5.8 square kilometers.

### The 1210 working face Monitoring data of surface subsidence points

**Figure 4:**
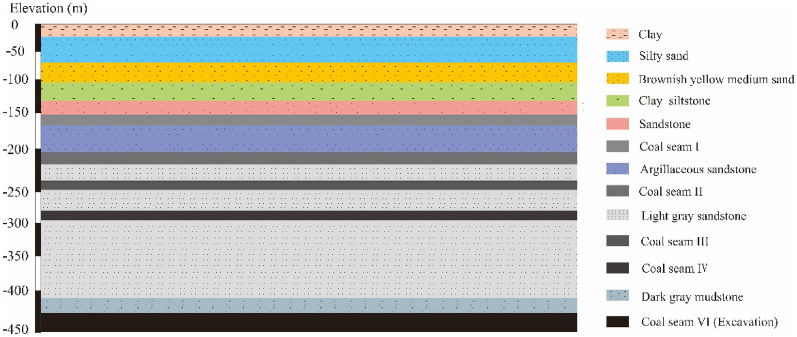
The Simplified geological section of Barapukuria Coal Mine.

**Figure 5:**
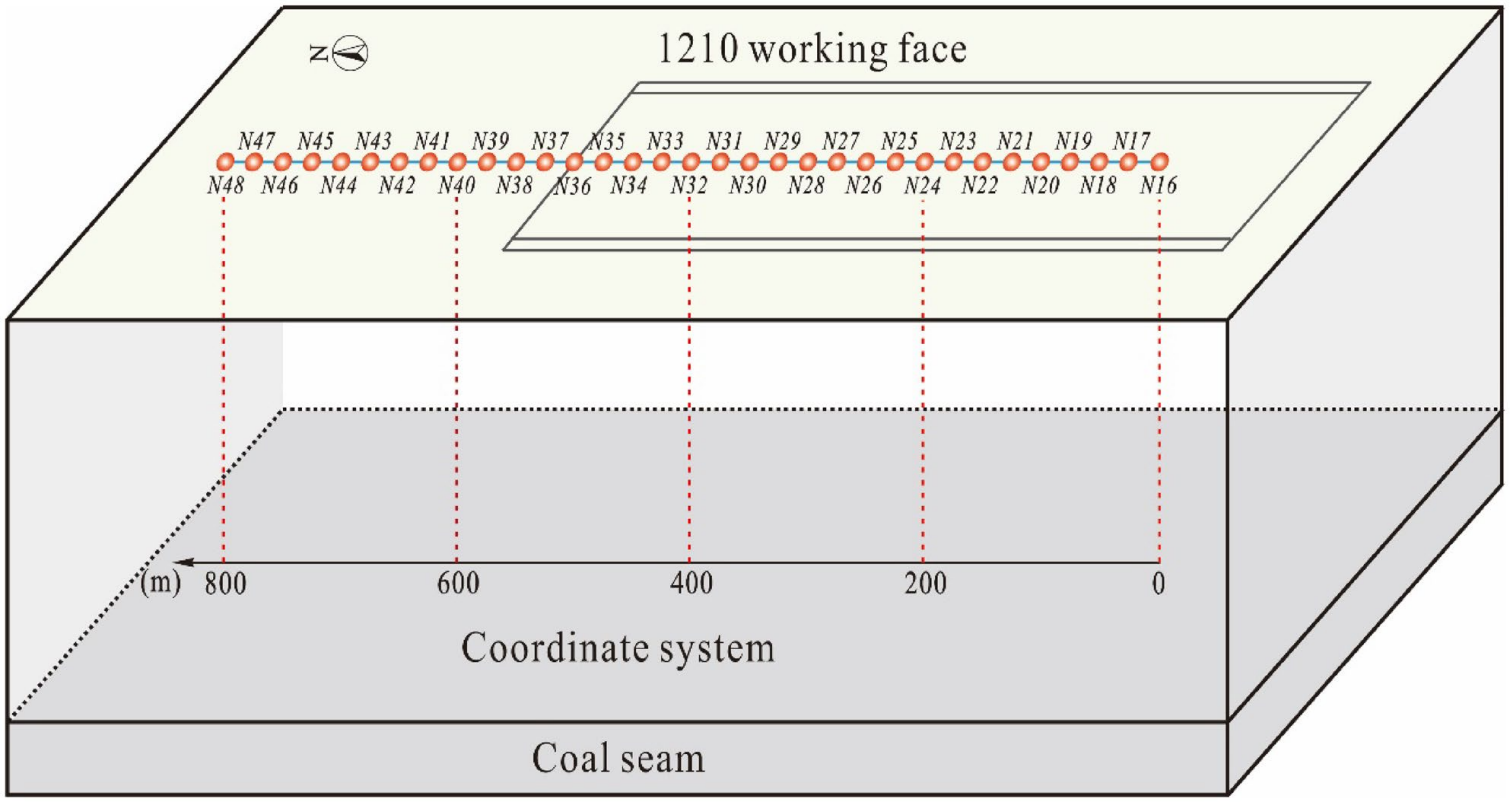
Comparison diagram of observation station of 1210 working face of Mumba Coal Mine (The letter N represents north–south direction).

**Figure 6:**
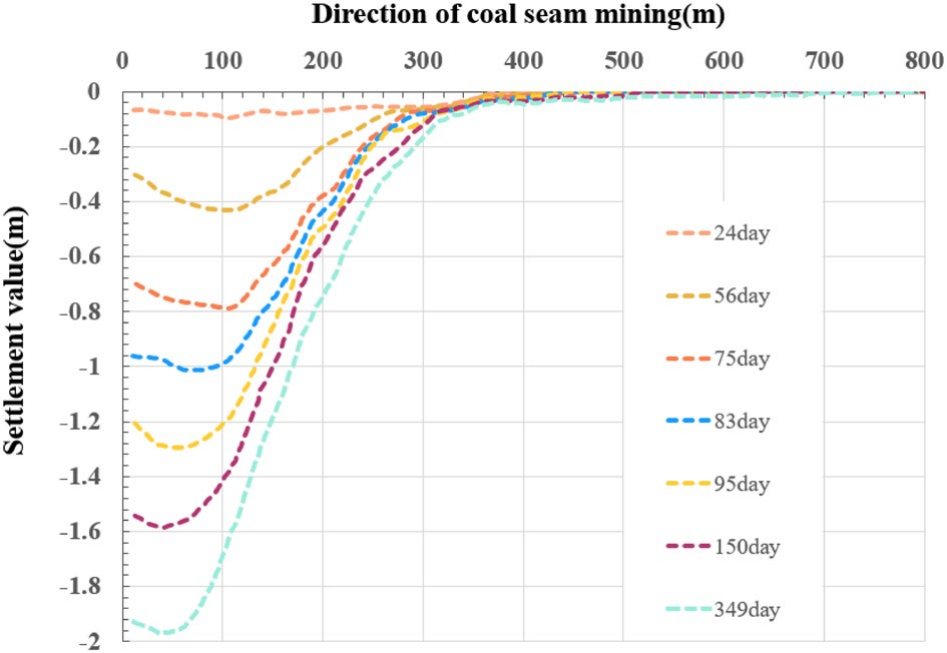
Monitoring data of surface subsidence of the strike principal section.

The coalfield stratum belongs to the Carboniferous-Permian, the 1210 working face is located in the south of the mine, and coal seam is horizontal with an average thickness of 5 m. The 1210 working face only laid a north–south direction observation line on the side of the open-off cut, with a total of 32 measuring points and with an average distance of approximately 25 m. The 1210 working face mining process lasted for 118 days. The average mining depth of the 1210 working face is 414 m, mining length is 349 m, and mining speed is 2.96 m/day. The 1210 working face can be regarded as a near-horizontal coal seam. The position of the mining area and a comparison diagram of the 1210 observation stations are shown in Figs. 4 and 5, respectively. Based on the monitoring data of the surface subsidence points shown in Fig. 6, the subsidence curves are in line with the law of surface subsidence, and the position of the maximum subsidence moves forward with the advance of the working face. At t = 118 days, the mining of 1210 working face was completed.

### Prediction of surface subsidence of the strike principal section

**Figure 7:**
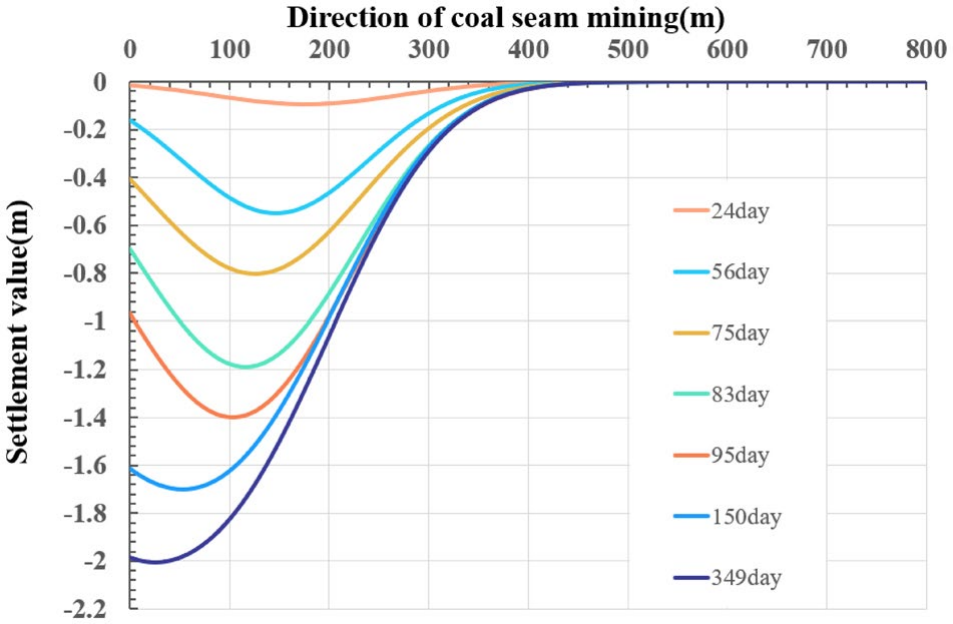
Prediction curve of surface subsidence of the strike principal section.

According to the empirical value of the prediction parameters of the mining area, with the help of Eqs. (7) and (14), we obtained the initial iterative value for least square fitting to obtain q = 0.71,$\tan \beta = 1.82$. Using the improved model, we get the ground subsidence results (Fig. 7). Figure 7 shows that these dashed and solid curves present the surface subsidence of the monitoring and prediction of surface points along the strike principal section, respectively. Because the monitoring data were affected by water on the ground, the origin of the coordinates was set at unaffected N16. The coal seam mining is carried out from monitoring point N24 to N16 in Fig. 5, starting at N24, until mining at 349 m. It is reasonable to show the subsidence value in the [200, 800 m] interval in Fig. 7, which can be considered to have an infinite boundary, but its influence range is limited. The state of the subsidence curve at seven different moments is provided in the prediction, and the position of the maximum subsidence point of the prediction curve is consistent with the theory. At T = 349 days, the maximum subsidence value of the simulation is 1.875 m, and the error with the measured data value of 1.968 is 4.72%. With the advance of the working face, the subsidence value of the ground point gradually increases, and the influence range of the surface subsidence basins gradually expands. The prediction curve shows that there is no flat base shape in the surface subsidence basin, which indicating that the strike direction of the working face does not reach full mining. This is consistent with the conclusion obtained from the measured data that the strike direction of the working face did not reach full mining. The prediction results of the subsidence using unimproved Knothe time function model is given in the Appendix, which can not predict the surface subsidence well with larger errors.

In both the monitoring data and the predicted value of the strike principal section, the overall subsidence rate first increased and then decreased, and the subsidence curve conformed to the law of the S curve. The average interval of the maximum subsidence value for each monitoring curve is 0.311 m, whereas the predicted curve is 0.317 m. After excavation, the number of days experienced for each increase in a certain subsidence value decreases and then increases, and the monitoring curve is in good agreement with the prediction.

### Simulation and prediction of subsidence basin

Based on the above prediction model described for surface subsidence, we solved Eqs. (11) and (14) with the help of MATLAB, and obtained the dynamic development of the surface subsidence basin. Here, *t* represents the duration from the moment of mining, and the total mining time was 118 days.

**Figure 8:**
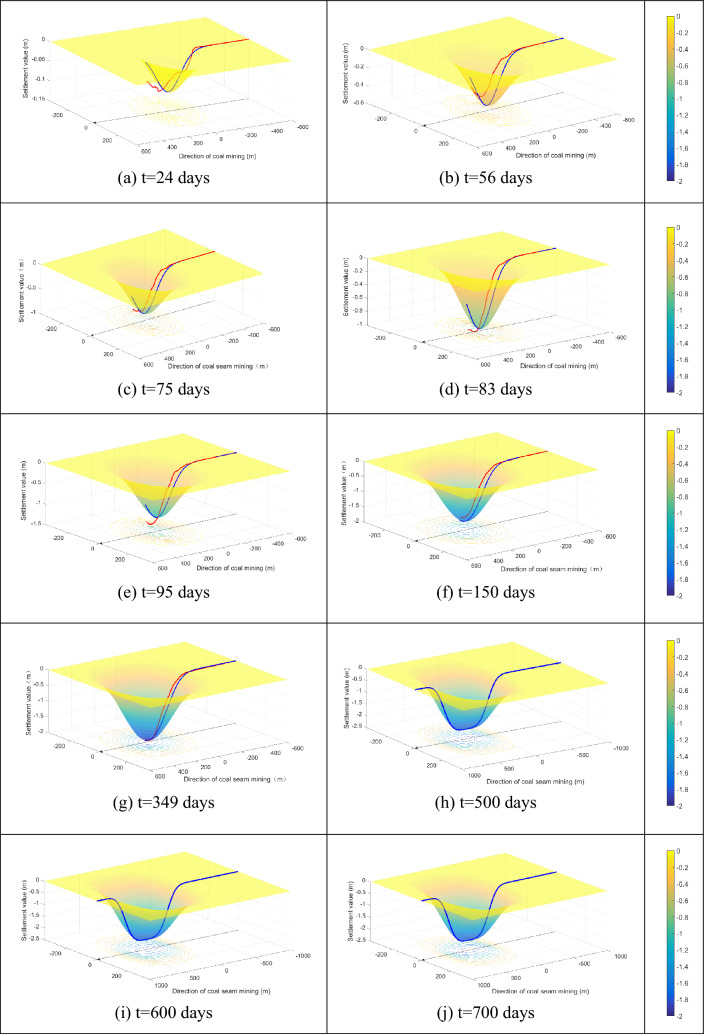
The subsidence basin at different moments (**a**–**j**) represent the subsidence basin at different time, the red and blue lines represent the monitoring data and prediction value of subsidence, respectively).

Figure 8 shows the subsidence basin prediction results at different moments, where the red and blue lines represent the monitoring data and prediction curves along the strike principal section, respectively.

Figure 8a shows the prediction result of the subsidence basin at t = 24 days, whose development trend of subsidence is the direction indicated by the arrow and the shape is funnel-shaped. The maximum subsidence values of the subsidence basin from the predicted and monitored data are − 0.101 m and − 0.105 m at t = 24 days, respectively. The error between the prediction model and measured data is − 3.23%. The horizontal projection of subsidence basin induced by mining can be approximately shown according to the contour lines, revealing the extension trend of subsidence.

Figure 8b–g show the different prediction results for the subsidence basin at t = 56, 75, 83, 95, 150, and 349 days. The maximum prediction values of the subsidence basin are − 0.543 m, − 0.854 m, − 1.189 m, − 1.400 m, − 1.702 m, − 2.005 m, respectively, and the extension trend of subsidence is along the arrow direction (mining direction). The maximum subsidence values of the monitoring data are respectively − 0.431 m, − 0.785 m, − 1.014 m, − 1.292 m, − 1.586 m, and − 1.968 m, and the red and blue lines represent the monitoring data and the subsidence prediction value of the strike principal section, respectively. The results show that the predicted values are slightly higher than the monitored data. The average error between the prediction value and the monitoring data is 9.49%, which indicates that our model can provide a good dynamic prediction of the subsidence caused by coal mining.

The surface subsidence continues to spread after mining, and with the help of our model, we can obtain some more predictions for the subsidence basin shown in Fig. 8h–j at different moments, and the bottoms of the subsidence basin become flat. The maximum values of the subsidence basin changed from − 2.077 m at 400 days to − 2.079 m at 700 days, indicating that the subsidence basin trends to be stable. The results in Fig. 8 show that the subsidence basin expands continuously and develops along the mining direction. The blue lines represent the predicted subsidence values for the strike principal section.

## Discussion

Surface subsidence caused by mining often produces subsequent disasters, the prediction of which may help to assess subsequent disasters. Based on the monitoring data of the Barapukuria mine, we developed a prediction model based on the optimized piecewise Knothe time function and prediction parameters from the actual monitoring results, which can determine the subsidence prediction curve of the strike principal section and subsidence basin at arbitrary points on the surface through simulations.

Our simulation shows that the prediction results and monitoring data are very similar, as shown in Fig. 8a–f. The blue lines prediction results do not completely coincide with the red lines monitoring data, which may be due to the following reasons: (1) The overlying lithology varies at different positions because the physical parameters of overburden strata constantly change, which directly affects the subsidence prediction. (2) For convenience, we ignored the influence of the inflection point offset distance. (3) There are some influences of mining-out on both sides in the actual mining process.

## Conclusion

In this study, we developed a time influence function to model surface subsidence caused by mining. These subsidence equations are implemented in MATLAB for the numerical prediction of surface subsidence in Barapukuria mining. The results of our simulation allow us to show how the subsidence basin extends at different times during the mining process. Our simulation shows that the predicted results agree well with the monitoring data. Our simulation provided the final shape of the subsidence basin. This may provide an early prediction of ground subsidence caused by mining.

The present simulation ignores the influence of the physical parameters of the overburden strata. In the next, we will give more consider the physical parameters of overburden strata and the inflection point offset distance to obtain a more accurate prediction model.

## Supplementary Information

Supplementary Information 1.

Supplementary Information 2.

## Data Availability

All data generated or analysed during this study are included in its supplementary information files and available from the corresponding author on reasonable request.
