# Supplementary material for: A time function-based prediction model of mining subsidence: application to the Barapukuria coal mine, Bangla
Source: Sci Rep. 2022 Nov 1;12:18433. doi: 10.1038/s41598-022-23303-9 (PMC9626622; doi:10.1038/s41598-022-23303-9)
Supplement: Supplementary file 1 — Supplementary Information 1. [file 41598_2022_23303_MOESM1_ESM.docx]

**Appendix**

The results of the prediction curve of the surface subsidence of the strike principal section using Knothe time function model are shown in Figure A1. The surface subsidence is almost zero at T=24 days. At T=56, 75, 83, 95, 150, and 349 days, the maximum prediction values of the subsidence are -0.14m, -0.39m, -0.51m, -0.70m, -1.41m, -1.84m, respectively. The maximum subsidence values of the monitoring data are respectively -0.43m, -0.79m, -1.01m, -1.29m, -1.59m, and -1.97m. Obviously, at T=56, 75, 83, and 95 days, there are large errors between the measured data and the maximum subsidence value of the simulation. At T=150 and 349 days, the errors between the measured data and the maximum subsidence value of the simulation are 11.20% and 6.60%, respectively.

Similarly, the subsidence basins at different moments using the Knothe time function model are simulated (Figure A2), which shows that there are large errors between the measured data and the predicted results at T=56, 75, 83, and 95 days. At T=150, and 349 days, the maximum prediction values of the subsidence are -1.51m and -1.96m, respectively, and the error between the measured data and the maximum subsidence value of the simulation are 5.01% and 0.193%, respectively. The optimized piecewise Knothe time function model used in this study is better than the Knothe time function model and has a better prediction performance for the selected mining area.


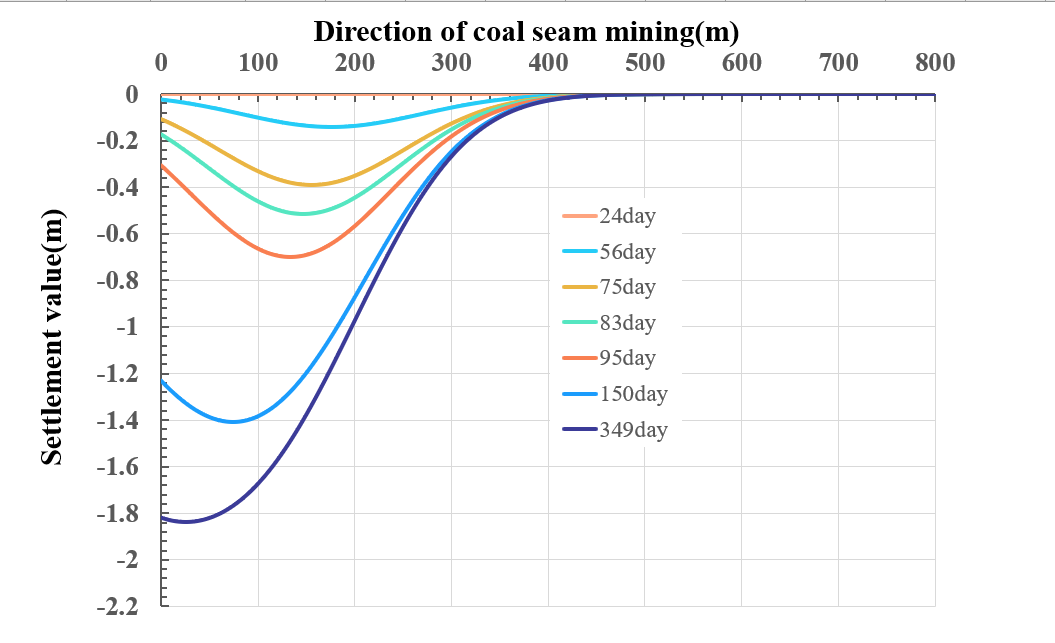


**Fig. A1. Prediction curve of surface subsidence of the strike principal section by using the Knothe time function**

| 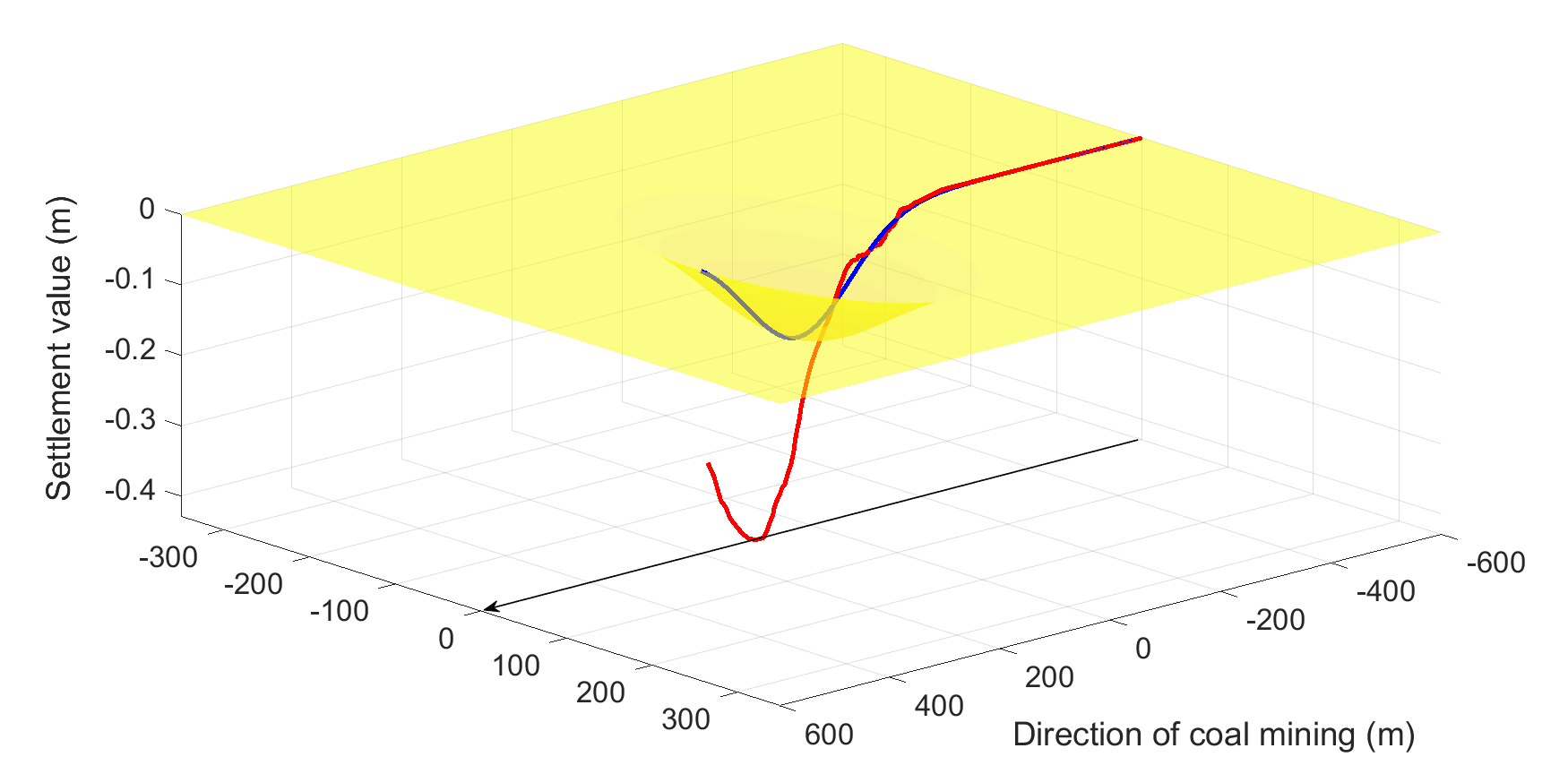  (a) T=56 days | 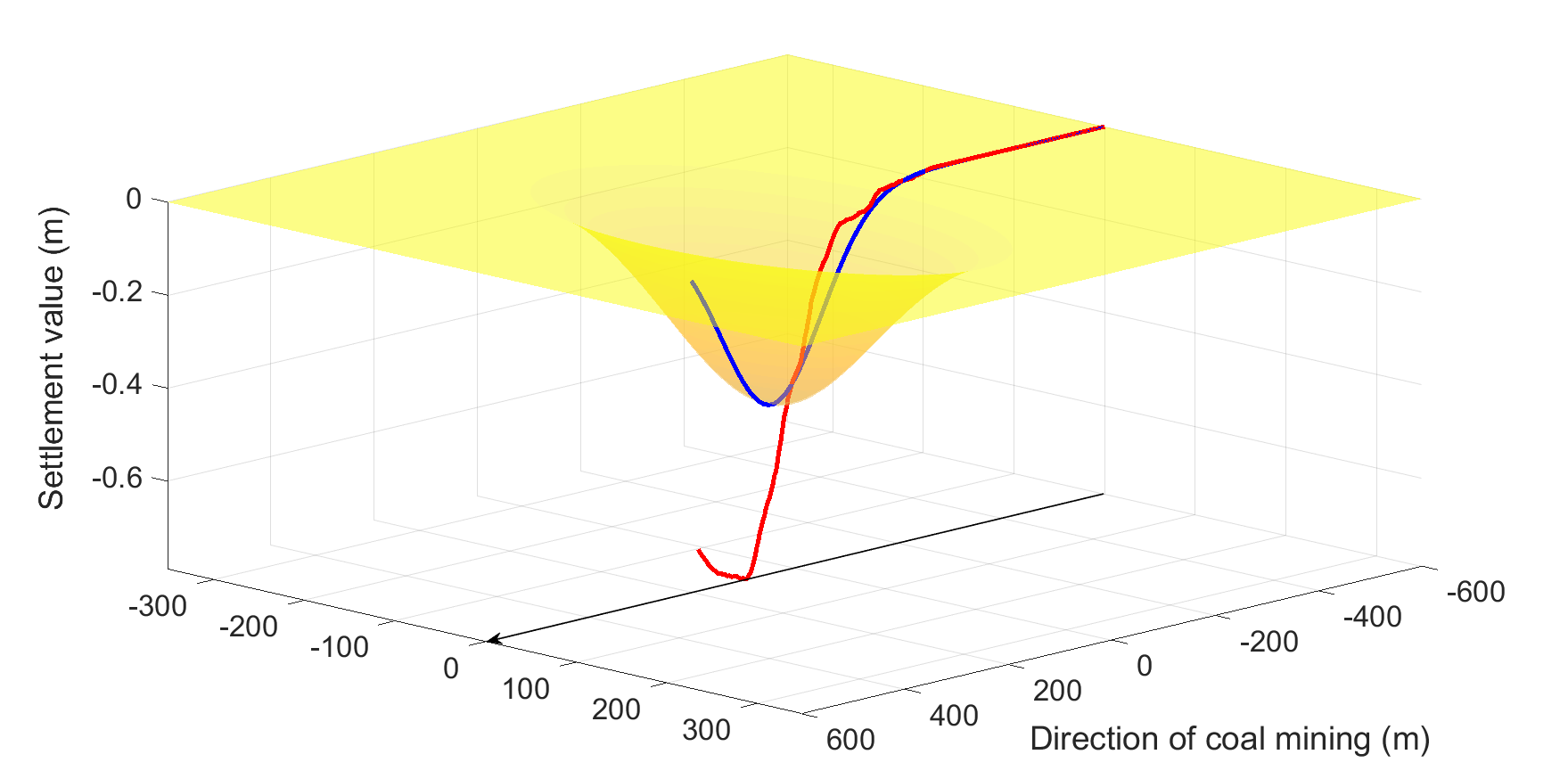  (b) T=75 days | 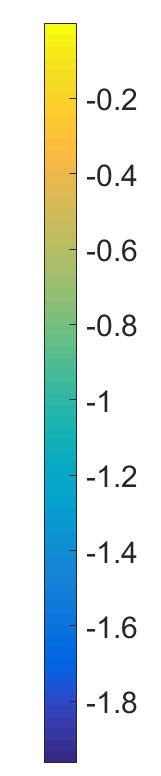 |
| --- | --- | --- |
| 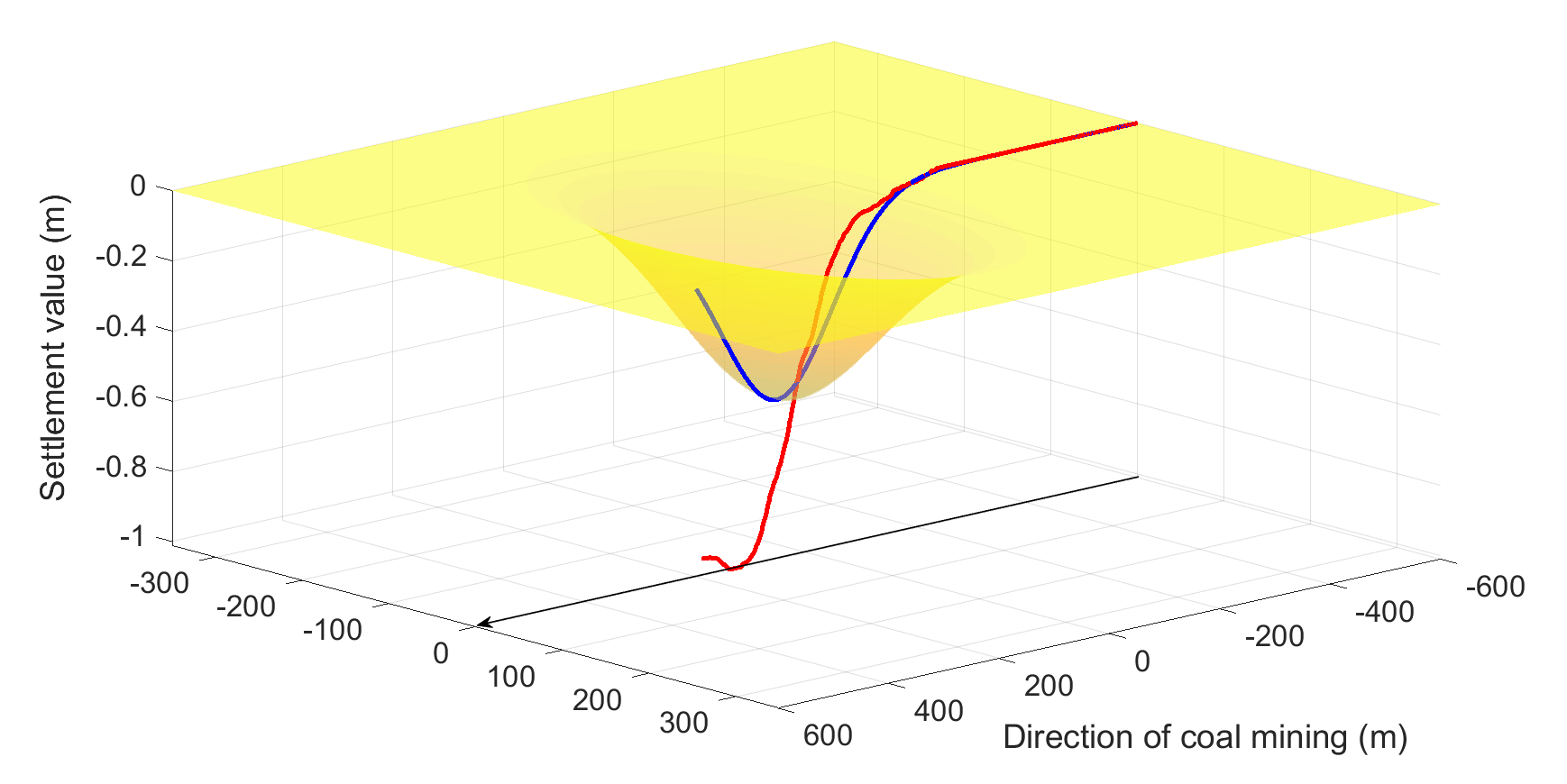  (c) T=83 days | 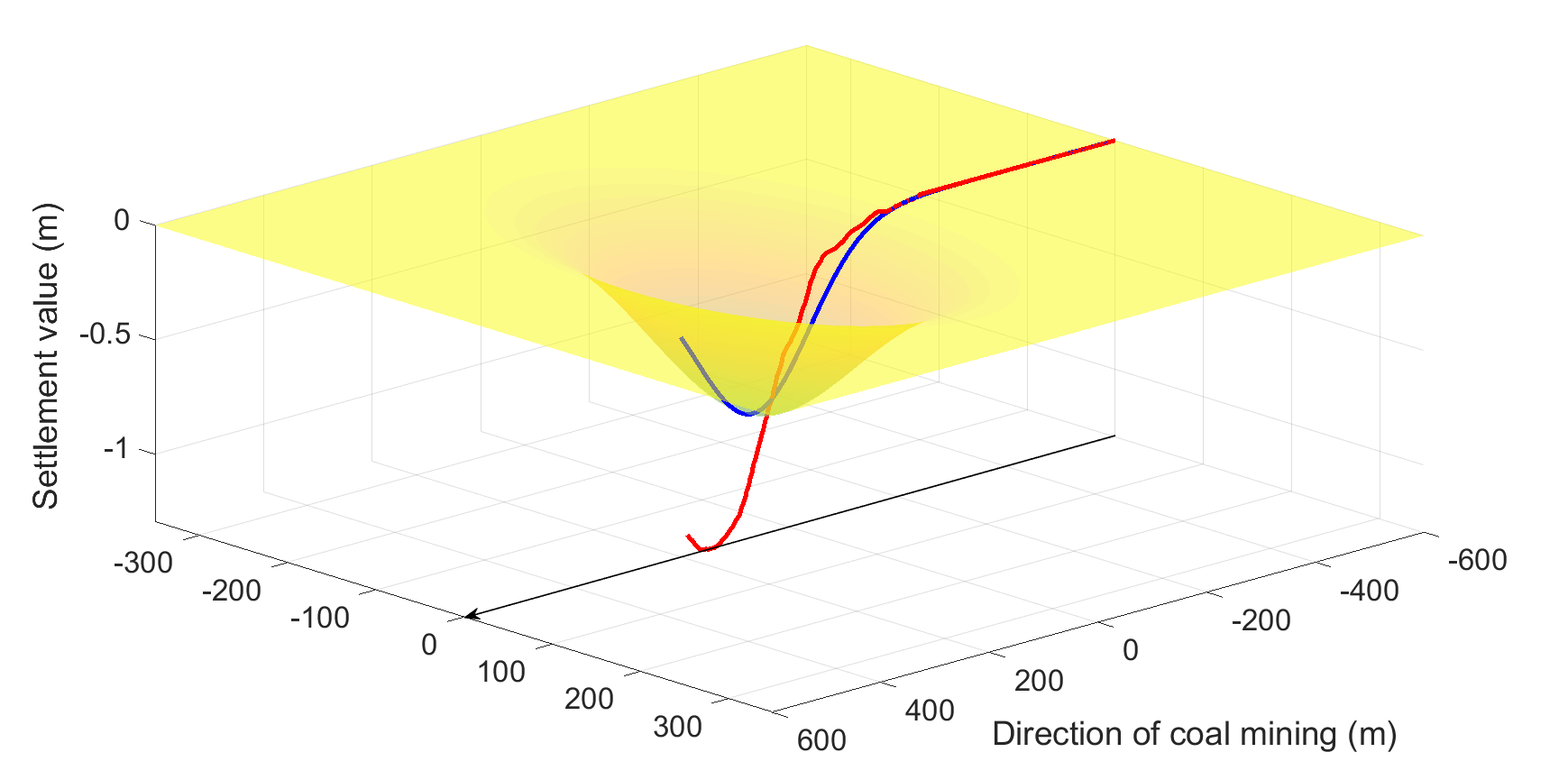  (d) T=95 days | 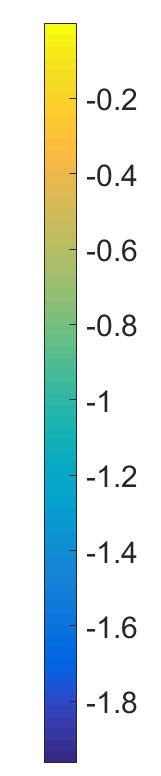 |
| 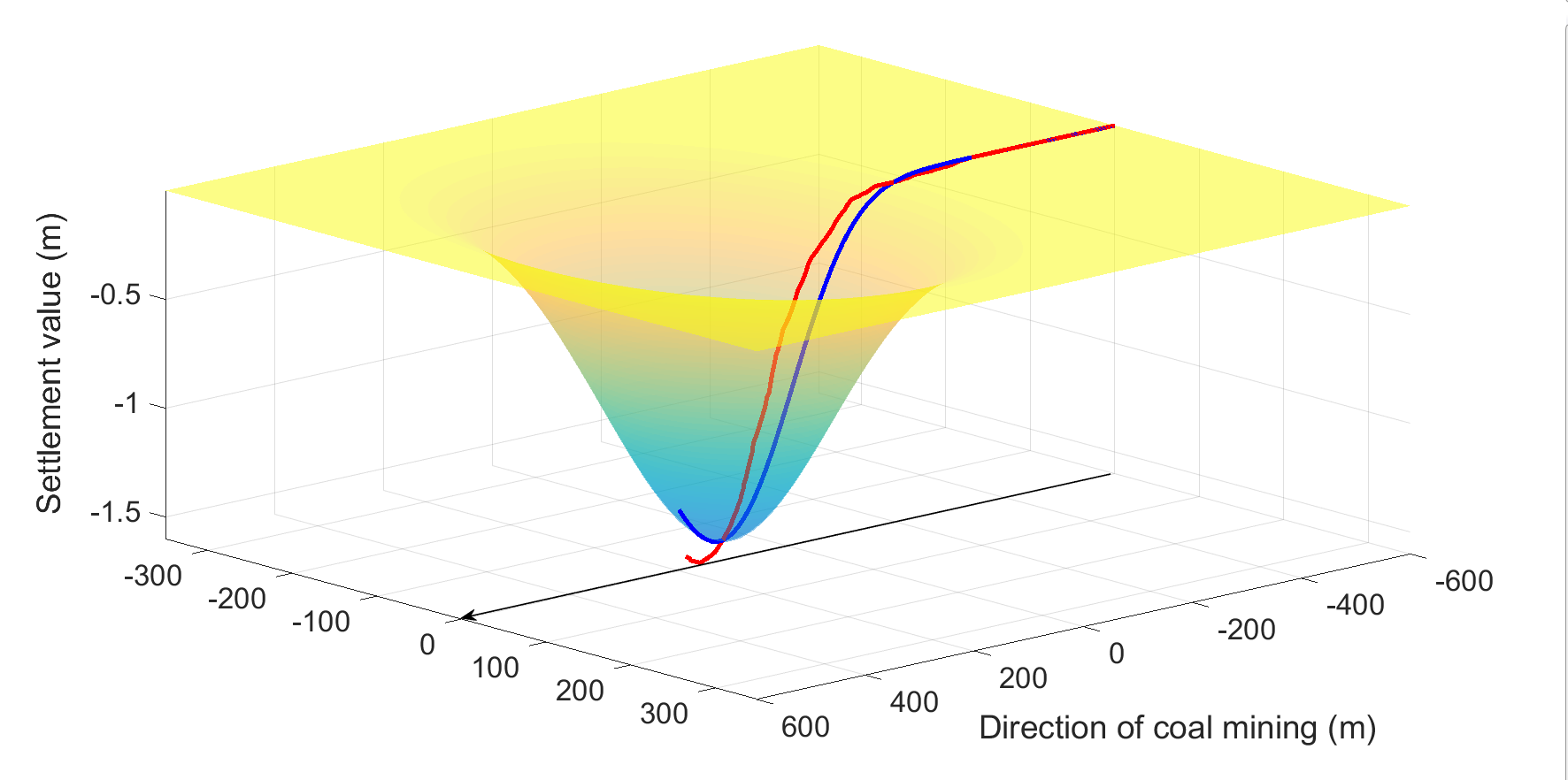  (e) T=150 days | 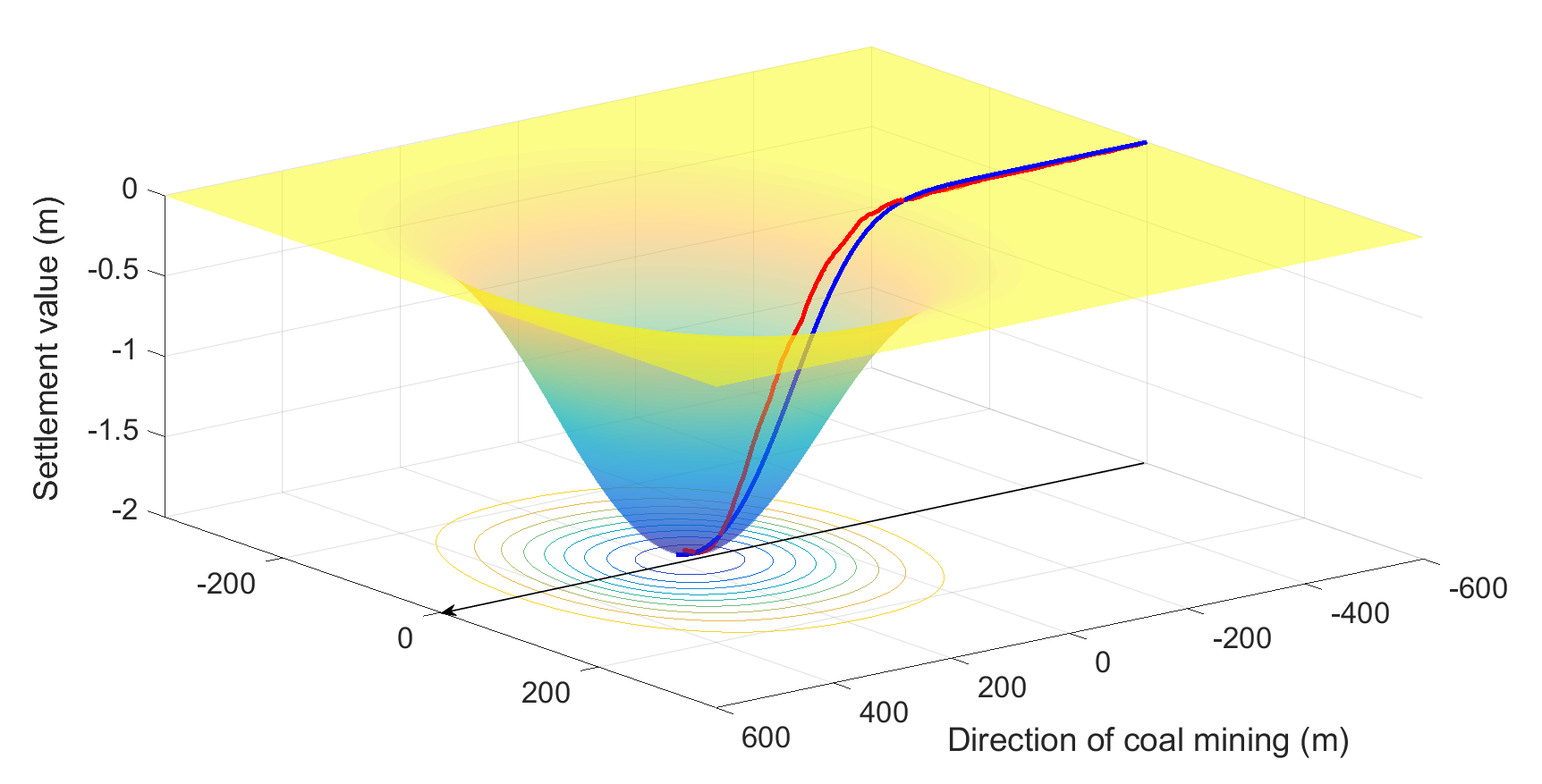  (f) T=349 days | 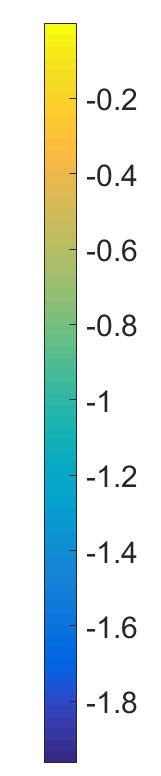 |

**Fig. A2. The subsidence basin at different moments by using Knothe time function ((a), (b), (c), (d), (e) and (f) represent the subsidence basin at different time, the red and blue lines represent the monitoring data and** **prediction value of subsidence, respectively)**
